# The Ubiquitination, Disaggregation and Proteasomal Degradation Machineries in Polyglutamine Disease

**DOI:** 10.3389/fnmol.2017.00078

**Published:** 2017-03-22

**Authors:** Samir R. Nath, Andrew P. Lieberman

**Affiliations:** ^1^Medical Scientist Training Program, University of Michigan Medical SchoolAnn Arbor, MI, USA; ^2^Cellular and Molecular Biology Graduate Program, University of Michigan Medical SchoolAnn Arbor, MI, USA; ^3^Department of Pathology, University of Michigan Medical SchoolAnn Arbor, MI, USA

**Keywords:** CAG polyglutamine disorder, ubiquitination, disaggregase machinery, Proteasome, chaperones

## Abstract

Polyglutamine disorders are chronic, progressive neurodegenerative diseases caused by expansion of a glutamine tract in widely expressed genes. Despite excellent models of disease, a well-documented clinical history and progression, and established genetic causes, there are no FDA approved, disease modifying treatments for these disorders. Downstream of the mutant protein, several divergent pathways of toxicity have been identified over the last several decades, supporting the idea that targeting only one of these pathways of toxicity is unlikely to robustly alleviate disease progression. As a result, a vast body of research has focused on eliminating the mutant protein to broadly prevent downstream toxicity, either by silencing mutant protein expression or leveraging the endogenous protein quality control machinery. In the latter approach, a focus has been placed on four critical components of mutant protein degradation that are active in the nucleus, a key site of toxicity: disaggregation, ubiquitination, deubiquitination, and proteasomal activity. These machineries have unique functional components, but work together as a cellular defense system that can be successfully leveraged to alleviate disease phenotypes in several models of polyglutamine toxicity. This review will highlight recent advances in understanding both the potential and role of these components of the protein quality control machinery in polyglutamine disease pathophysiology.

## Introduction

Polyglutamine (polyQ) diseases encompass nine untreatable and invariably fatal neurodegenerative diseases associated with protein misfolding and aggregation. The cause of this family of disease is expansion of a glutamine tract within widely differing proteins, leading to gain of function effects and a shared phenotype of adult-onset progressive neurodegeneration. There are no FDA approved disease modifying treatments for any polyQ disease despite their well-established genetic causes, carefully documented clinical history, and the availability of excellent genetic models that recapitulate aspects of the disease phenotype (Chua and Lieberman, 2013). Work in these model systems over the last several decades has highlighted downstream toxic effects in a large number of pathways, including those regulating gene expression, axonal transport, mitochondrial function, and energy metabolism (Mhatre et al., 1993; Chamberlain et al., 1994; Kazemi-Esfarjani et al., 1995; Irvine et al., 2000; McCampbell et al., 2000; Lieberman et al., 2002; Szebenyi et al., 2003; Morfini et al., 2006; Ranganathan et al., 2009; Kemp et al., 2011; Giorgetti et al., 2016; Rocchi et al., 2016). These observations suggest that targeting any single pathway for therapeutic benefit may be incomplete and ineffective. As a consequence, strategies to rid cells of the mutant proteins have attracted considerable recent attention. Both reducing expression of the mutant protein using antisense oligonucleotides (Lieberman et al., 2014; Sahashi et al., 2015; Giorgetti et al., 2016) and enhancing degradation of the mutant protein (Sittler et al., 2001; Adachi et al., 2003, 2007; Tokui et al., 2009; Wang et al., 2013; Silva-Fernandes et al., 2014) lead to phenotypic rescue of multiple polyQ disease models, establishing removal of the mutant protein as a viable therapeutic strategy. This review will focus on pathways involved in mutant protein degradation, highlighting pathophysiologic changes, and possible therapeutic targets within the fields of ubiquitination, deubiquitinating enzymes, protein disaggregation, and proteasomal activity. Though misfolded proteins are also degraded through autophagy, several excellent recent reviews have detailed the contribution of this pathway to their clearance and potential strategies for therapeutic manipulation (Martin et al., 2015; Rusmini et al., 2015, 2016).

## Ubiquitinating enzymes (E3s) in polyQ diseases

A wealth of studies implicates the ubiquitination machinery as a potential therapeutic target in polyglutamine diseases. These enzymes serve a key role in targeting proteins for degradation both by the proteasome and autophagy (Pratt et al., 2015; Rusmini et al., 2016). In the last decade, a great deal of focus has been placed on C-terminal Hsp70-interacting protein (CHIP), which acts as both a co-chaperone and an E3 ubiquitin ligase for misfolded proteins and plays a key role in facilitating their degradation (Pratt et al., 2015).

**Figure 1:**
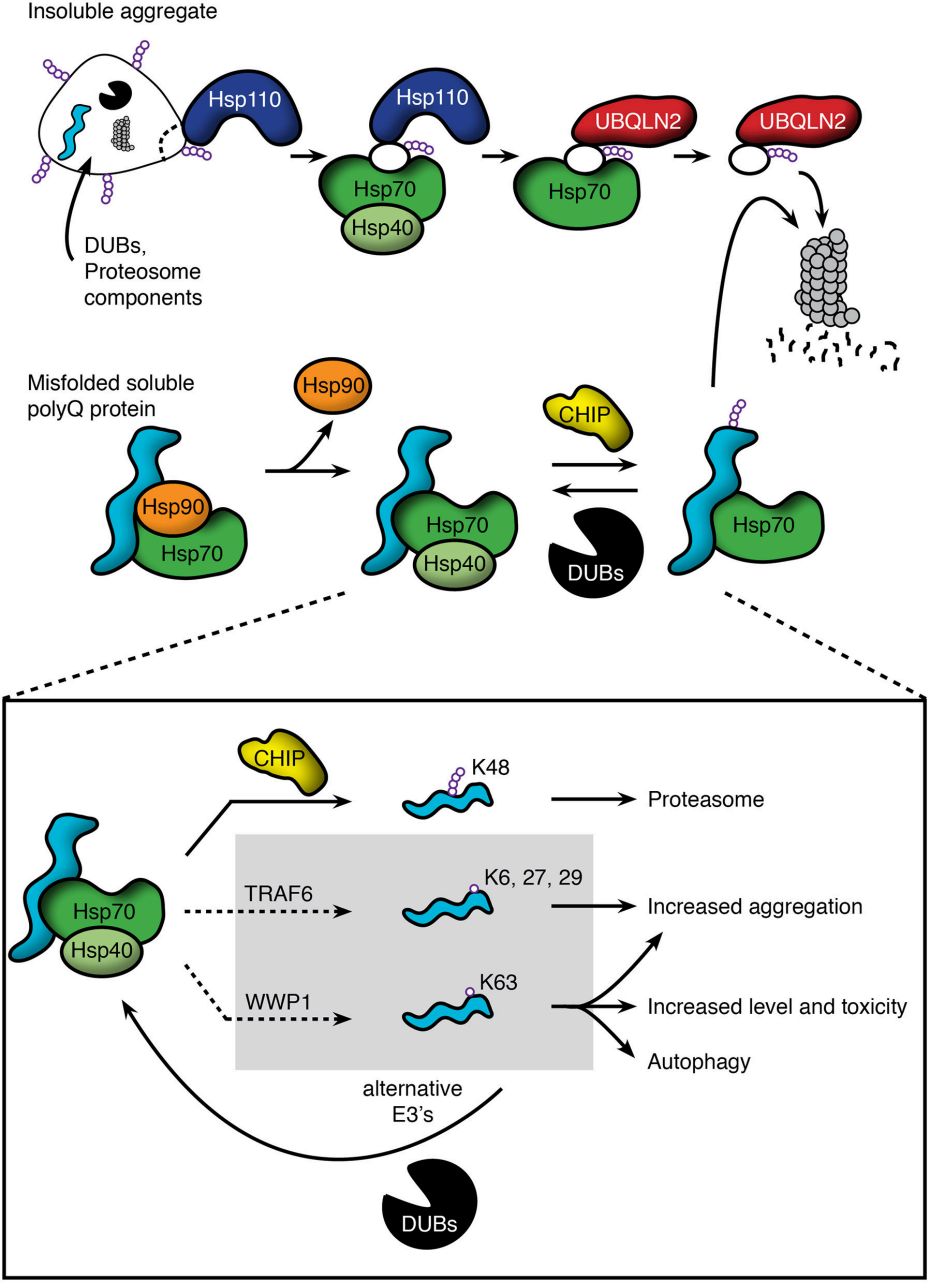
**Pathways regulating polyQ protein degradation through the proteasome. (Top)** Insoluble protein aggregates are recognized by Hsp110, which acts to disaggregate and remove misfolded proteins. These substrates are then passed to the Hsp70/40 complex and subsequently bound by the ubiquitin-associated domain of UBQLN2. UBQLN2 traffics substrates to the proteasome, which recognizes UBQLN2's ubiquitin-like domain. **(Middle)** PolyQ proteins that are clients of the Hsp90/Hsp70 based chaperone machinery include the androgen receptor and huntingtin. Soluble species interact with the chaperone machinery, but release Hsp90 upon misfolding. This allows substrate bound Hsp70/Hsp40 to recruit E3 ligases, such as CHIP, to promote polyubiquitination and proteasomal degradation. This action is opposed by deubiquitinating enzymes (DUBs), which can remove the polyubiquitin chains and send soluble polyQ proteins back into the cellular pool. **(Bottom)** Though E3 ligases such as CHIP promote K48-linked polyubiquitination and proteasomal degradation, emerging evidence shows that other E3's can ubiquitinate using alternative lysine residues, leading to impairment of degradation, increased aggregation, and/or degradation through alternative pathways such as autophagy. For these ligases, DUBs may act in a beneficial manner to return polyQ proteins to the pool of proteins that can be polyubiquitinated through K48 linkages and degraded through the proteasome.

CHIP's potential as a therapeutic target centers on its ability to promote ubiquitination and proteasomal degradation of client proteins of the Hsp90/Hsp70 based chaperone machinery (Zhou et al., 2003; Pratt et al., 2015; Chung et al., 2016). In this machinery, Hsp90 and Hsp70 bind to client proteins in their native or near native conformations to regulate many aspects of proteostasis. The binding of Hsp90 to client proteins regulates hydrophobic protein clefts. Disease-causing mutations either destabilize clefts or introduce a second site of inherent instability that also binds chaperones. Interaction with Hsp90 stabilizes hydrophobic clefts; as mutant proteins unfold, interaction with Hsp90 is lost, leaving client-bound Hsp70 to recruit CHIP to promote ubiquitination (Pratt et al., 2015; Figure 1, middle). Notably, truncated fragments of disease-causing proteins that lack hydrophobic clefts present in the full-length protein may fail to interact with the Hsp90/Hsp70 based machinery. This is the case for an amino-terminal fragment of the polyQ androgen receptor that lacks the ligand-binding domain. The consequence is that degradation of this artificial construct is handled differently from the full-length protein and is largely mediated by autophagy (Wang et al., 2010).

In our model of chaperone machinery protein triage, Hsp70 binds client proteins and plays a critical role by recruiting CHIP to misfolded substrates, which in turn promotes ubiquitination and degradation of these proteins. The recruitment of CHIP may be influenced by post-translational modifications of Hsp70, such as phosphorylation of a serine or threonine residue near the EEVD motif (VanPelt and Page, 2017). CHIP's ability to recognize and ubiquitinate Hsp90 clients depends on the binding cleft in the full-length protein. When CHIP substrates, such as nNOS (Peng et al., 2004), ErbB2 (Zhou et al., 2003), and estrogen receptor (Fan et al., 2005), are manipulated by site-specific inhibitors within the ligand binding cleft, ubiquitination and degradation are increased (Wijayaratne and McDonnell, 2001; Citri et al., 2002; Peng et al., 2009), supporting the crucial role of the ligand binding cleft as a recognition motif for the Hsp70-CHIP complex.

Due to its ability to ubiquitinate client proteins, CHIP has been studied extensively in polyQ diseases. In Huntington disease models, CHIP over-expression suppresses aggregation and toxicity of polyQ huntingtin both in non-neuronal cells and primary neurons. In contrast, haploinsufficiency of CHIP leads to accelerated disease in mice over-expressing polyQ huntingtin (Miller et al., 2005). CHIP drives degradation of polyQ huntingtin by promoting ubiquitination and proteasomal degradation of the mutant protein, an action CHIP can also perform on ataxin-3, the causative protein in spinocerebellar ataxia type 3 (SCA3; Jana et al., 2005). Importantly, ataxin-3 deubiquitinates CHIP, terminating CHIP-substrate interaction; polyQ expansion of ataxin-3 increases its affinity for CHIP and decreases CHIP levels in SCA3 mice, suggesting a surprising role for coordinated regulation of CHIP and ataxin-3 as well as dysregulation of this process in SCA3 (Scaglione et al., 2011). In spinal and bulbar muscular atrophy (SBMA), over-expression of CHIP leads to phenotypic rescue of a transgenic mouse model over-expressing polyQ androgen receptor. This degradation is selective for the mutant over the wild type receptor, indicating a preference for misfolded substrates (Adachi et al., 2007). Recently, CHIP was identified as a causative gene for autosomal recessive cerebellar ataxia, further implicating it as a crucial protein in preventing neurodegeneration and regulating neuronal homeostasis (Shi et al., 2013).

There is likely functional redundancy between CHIP and other chaperone-dependent E3 ligases. In support of this notion, CHIP deletion in cells had no effect on degradation of the polyQ androgen receptor or the glucocorticoid receptor, which are both known CHIP substrates (Morishima et al., 2008). To date no broadly overlapping E3s have been identified, raising the possibility that multiple E3 ligases are up-regulated in the setting of CHIP deletion.

Whereas E3s such as CHIP have been shown to play a protective role in polyQ disease, not all E3s fit this mold (Figure 1, bottom). TRAF6 is an E3 that is present in polyQ huntingtin aggregates and ubiquitinates polyQ huntingtin using lysine residues K6, K27, and K29 within ubiquitin. In cellular models, this ubiquitination leads to increased aggregation of the polyQ protein without a change in wild type huntingtin localization (Zucchelli et al., 2011). WWP1 is another E3 that colocalizes with polyQ huntingtin aggregates, and is up-regulated in both mice and N2a cells expressing polyQ huntingtin. Interestingly, over-expression of WWP1 leads to increased polyQ huntingtin levels and toxicity. The toxic effects of WWP1 have been attributed to its ability to ubiquitinate polyQ huntingtin at a K63 residue, leading to reduced proteasomal degradation of the mutant protein as well as increased toxicity in mouse and cellular models (Lin et al., 2016).

Together, this evidence provides a framework in which E3s such as CHIP function together with molecular chaperones to both recognize and ubiquitinate misfolded protein substrates, leading to their proteasomal degradation. However, E3s must be considered on a case by case basis, as growing evidence supports the idea that non-K48 linked ubiquitination can have opposite and regulatory effects on cargo. We suggest that enhancing activity of canonical E3s such as CHIP while inhibiting other E3s such as TRAF6 and WWP1 may have cooperative therapeutic benefits to promote polyQ protein degradation.

## Deubiquitinating enzymes

To counteract the activity of ubiquitin ligases, a system of checks and balances has been identified and characterized in the form of a network of deubiquitinating enzymes (DUBs). Over 90 DUBs have been identified within the human genome, each belonging to one of seven distinct families. These enzymes play diverse roles including altering protein function and impacting proteasomal degradation.

Recently, DUBs have emerged as attractive therapeutic targets in polyglutamine disorders. By removing ubiquitin tags from substrates, DUBs can delay proteasomal degradation. In contrast, their inhibition enhances substrate degradation, demonstrating a pivotal role in diseases caused by misfolded protein accumulation (Hanna et al., 2006; Lee et al., 2010). It is well-established that DUBs interact with polyQ proteins, such as ataxin-1 (Hong et al., 2002), ataxin-3 (He et al., 2016), huntingtin (Hyrskyluoto et al., 2014), and androgen receptor (Dirac and Bernards, 2010; Burska et al., 2013; Schrecengost et al., 2014; Chen et al., 2015). However, polyQ proteins can also sequester DUBs, leading to their inactivation. For example, polyQ ataxin-7 sequesters USP22 into aggregates and inhibits its DUB activity (Yang et al., 2015). DUBs can also regulate polyQ proteins. USP19b over-expression leads to an Hsp90-dependent increase in levels of wild type and polyQ ataxin-3 as well as polyQ Htt (He et al., 2016).

One DUB that has attracted considerable attention is USP14, which binds ubiquitinated substrates and facilitates opening of the proteasomal gate, allowing for selective and efficient substrate degradation (Peth et al., 2009). Modulating the activity of this DUB has significant effects on substrate degradation. Over-expression of USP14 reduces polyQ huntingtin aggregation and protects against cell degeneration by inhibiting IRE1α phosphorylation and blocking ER stress in PC6.3 cells (Hyrskyluoto et al., 2014). In contrast, Lee et al. have shown that over-expression of USP14 leads to buildup of both wild type and polyQ ataxin-3, while pharmacologic inhibition of USP14 leads to enhanced polyQ ataxin-3 and tau clearance in MEFs (Lee et al., 2010). These contrary findings may be explained by cell line or protein specific differences, or effects on total ubiquitin pools in these cells as a result of modulating global deubiquitination. Nonetheless, they underscore the physiological importance of the USP family of DUBs.

DUBs not only modify the degradation and steady state levels of polyQ proteins, but also influence the function of their wild type counterparts. DUBs have been intensively studied in cancer, resulting in the identification of a number of enzymes that modulate activity of cancer-causing genes. In particular, androgen receptor, mutations in which cause both prostate cancer and SBMA, is regulated in diverse ways by several DUBs. USP26 binds to androgen receptor through three nuclear receptor interaction motifs, and knockdown of USP26 leads to decreased receptor activity in LnCaP and HEK293 cells (Dirac and Bernards, 2010). USP10 is required for androgen receptor activated transcription of PSA and KLK3, and this role has been attributed to USP10's hormone-induced deubiquitylation of chromatin around androgen regulated genes (Draker et al., 2011). USP7 shows androgen-dependent association and deubiquitination of androgen receptor. This deubiquitination is essential for the androgen responsive binding to chromatin and subsequent transcriptional activation, a function that can also be performed by USP12 (Burska et al., 2013; Chen et al., 2015). The role of these enzymes in modulating polyQ AR function and toxicity has not yet been explored. As the physiologic function of many other polyQ disease causing proteins is not fully understood, how DUBs may affect their function remains an emerging field of investigation.

Underscoring the importance of DUBs to polyQ disease is SCA3, a disorder caused by expansion of a polyQ tract within the DUB ataxin-3. This DUB has many important cellular targets including p53. Interestingly, lack of polyQ ataxin-3's catalytic activity leads to increased toxicity in models of SCA3 (Warrick et al., 2005; Todi et al., 2010). This effect may be due to loss of protein function or buildup of the toxic protein, which is less readily recognized by p97 and inefficiently degraded by the proteasome (Todi et al., 2007). Overall, this work on ataxin-3 and other DUBs shows that this family of proteins serves critical roles in modulating the function, localization and degradation of polyQ proteins, and represents an exciting new therapeutic target.

## Disaggregation

Downstream of aggregation, a mechanism of disaggregation by which cells dismantle inclusions and degrade misfolded proteins has been characterized as functionally relevant to polyQ diseases (Figure 1, top). A number of key components of the mammalian disaggregase machinery have been identified including heat shock protein 110 (Hsp110; yeast Hsp104), ubiquilin-2 (UBQLN2), heat shock protein 70 (Hsp70), and heat shock protein 40 (Hsp40/DnaJ).

Hsp110 is a heat shock inducible protein first shown to play a key role in stress response and survival, a function that is conserved from bacteria to yeast and mammalian cells (Sanchez and Lindquist, 1990; Sanchez et al., 1992; Weber-Ban et al., 1999). Hsp110 neither promotes proteolysis nor protects the folded state of denatured luciferase, but mediates resolubilization of the aggregated protein (Parsell et al., 1994). The ability to disaggregate denatured luciferase is dependent on ATP and an intact nucleotide-binding site, suggesting a role for ATP hydrolysis in disaggregation. This activity is also dependent on Ydj1, an Hsp40 which promotes ATP hydrolysis, and Ssa1, a yeast Hsp70, implicating other members of the heat shock protein family in disaggregation (Glover and Lindquist, 1998).

Hsp110 has been implicated in blocking aggregation and toxicity of polyQ proteins. Over-expression of yeast Hsp104, an Hsp110 homolog, reduces aggregation of 72Q and 103Q huntingtin, and surprisingly, deletion of Hsp104 eliminates aggregation almost entirely (Krobitsch and Lindquist, 2000). This finding parallels the effects of Hsp104 on the yeast prion [PSI+]. These data support a model in which low levels of Hsp110 play a role in the recognition and seeding of prion-like aggregates, while high levels lead to recognition and solubilization of aggregated proteins (Chernoff et al., 1995; Patino et al., 1996). The role of Hsp110 in disaggregation of polyQ proteins has also been demonstrated in *C. elegans* expressing polyQ-GFP. In this system, over-expression of Hsp110 led to reduced aggregation and rescue of toxicity (Satyal et al., 2000). Further, over-expression of yeast Hsp104 and bacterial GroEL in Cos-7 and PC-12 cells reduced aggregation of polyQ huntingtin, supporting conserved disaggregase activity in mammalian cells (Carmichael et al., 2002). Using sequential mass spectrometry in *S. cerevisiae*, a model of chaperone binding has started to emerge. Hsp70 and Hsp90 appear to function in initial recognition of polyQ proteins, and are released prior to aggregate maturation. Subsequent aggregate formation occurs prior to Hsp104 interaction, suggesting a preference of Hsp104 for the aggregated species (Walter et al., 2011).

In the last several years a new addition to the disaggregase machinery has been characterized, ubiquilin-2 (UBQLN2). UBQLN2, also known as hPLIC2, is encoded by an intronless gene and contains both a ubiquitin-like domain and a ubiquitin-associated domain (Deng et al., 2011). Early studies showed that the ubiquitin-associated domain interacts with polyubiquitin chains on substrates, and the ubiquitin-like domain served to bring these substrates to and bind the proteasome. However, these studies also showed that over-expression of UBQLN2 interfered with proteasomal degradation, increasing the half-life of substrates such as p53 and IκBα (Kleijnen et al., 2000, 2003). Several subsequent studies have corroborated impaired proteolysis downstream of UBQLN2 over-expression (Chen and Madura, 2002; Raasi and Pickart, 2003; Massey et al., 2004). This relationship between substrate recognition and proteasomal inhibition suggests that UBQLN2 function is best studied at endogenous levels (Verma et al., 2004). Consistent with these findings, Hjerpe et al. recently demonstrated a key role for UBQLN2 in the disaggregase machinery. Knockdown of UBQLN2 led to reduced survival and inability to clear insoluble ubiquitin aggregates after heat shock. Importantly, the clearance of insoluble aggregates was shown to be dependent on previously mentioned members of the disaggregase machinery including Hsp70 and Hsp110. The authors also showed that UBQLN2 colocalizes with polyQ huntingtin aggregates, and reduction of UBQLN2 led to increased aggregation (Hjerpe et al., 2016).

The above evidence supports a model in which Hsp70/Hsp110 acts to recognize and solubilize protein aggregates. UBQLN2 associates with client-bound Hsp70 and assists in shuttling aggregated components to the proteasome. One key remaining question is whether disaggregation alleviates toxicity in organismal models of polyglutamine disease, which will identify the importance of the disaggregation system for therapeutic strategies. Understanding how to therapeutically manipulate the disaggregase machinery is a worthwhile endeavor likely to lead to a better understanding of pathophysiology.

## Proteasome dysfunction in polyglutamine diseases

A wealth of evidence has linked the proteasome to several polyQ diseases, both as a therapeutic target and as a dysfunctional pathway downstream of mutant polyQ proteins. The 20S proteasome was first shown to colocalize with aggregates of polyQ ataxin-1 in both patients and transgenic mice. This same colocalization was later found in human SCA3 tissue, a mouse model expressing polyQ ataxin-7, HeLa cells expressing polyQ androgen receptor, and mice over-expressing truncated polyQ androgen receptor (Cummings et al., 1998; Chai et al., 1999; Stenoien et al., 1999; Yvert et al., 2000; Abel et al., 2001). Interestingly, in human brain sections from SCA3 patients, only the 11S and 19S regulatory particles were found in inclusions, suggesting differential recruitment of proteasomal components for misfolded aggregates (Schmidt et al., 2002). Though proteasomes seem to colocalize with the majority of polyglutamine inclusions, they are not completely trapped, as some exchange of proteasomes within inclusions was observed using FRAP (Stenoien et al., 2002).

The proteasome also shows a preference for degrading misfolded polyQ proteins over their wild type counterparts, likely due to efficient targeting of mutant proteins for degradation. In MN1 cells expressing either 24Q or 65Q androgen receptor, the mutant protein demonstrates significantly reduced half-life, and this degradation is inhibited by the proteasome inhibitor lactacystin (Lieberman et al., 2002). Similarly, in cells expressing 20Q and 76Q huntingtin, proteasome inhibition leads to greater buildup of the toxic, N-terminal polyQ huntingtin compared to wild type, and dramatically more buildup of the polyQ protein following proteasome inhibition compared to autophagy inhibition (Li et al., 2010).

While the proteasome has been implicated in polyQ protein degradation, studies have also shown impaired function in several disease models. In mice over-expressing N-terminal polyQ huntingtin, altered proteasome localization to aggregates was accompanied by an increase in half-life of the proteasome substrate p53 (Jana et al., 2001). In cells over-expressing N-terminal polyQ huntingtin or ΔF508 CFTR (cystic fibrosis transmembrane conductance regulator), cells with aggregates had a significant impairment in proteasome activity compared to those lacking aggregates when function was measured by clearance of the fluorescent reporter protein GFPu (Bence et al., 2001). Notably, the tight association of aggregation with the accumulation of GFPu was not reproduced in a mouse model of Huntington disease (Bett et al., 2009), raising the possibility that soluble misfolded species of the mutant protein might impair proteasome function in some model systems. When SH-SY5Y cells were stably transfected with polyQ-GFP, there was impaired ability of the proteasome to compensate for heat shock-induced stress (Ding et al., 2002). In cerebellar neurons expressing polyQ ataxin-7, proteasome function was significantly diminished, and this led to cytosolic accumulation and impaired activity of NF-κB, culminating in increased activation of caspase-9 (Wang et al., 2007). The impairment in proteasome function downstream of polyQ proteins may be indirect, as studies in yeast and mammalian cells have demonstrated that accumulated substrates are less likely to be shuttled to the proteasome, potentially due to dysregulation of the key proteasome chaperone Sis1p or its mammalian ortholog DnaJB1 (Park et al., 2013). Together, this evidence suggests a role for proteasome impairment in models of polyQ diseases.

Though cellular data has been very consistent in demonstrating proteasome dysregulation, *in vivo* studies have lacked consistent results (Bowman et al., 2005; Bennett et al., 2007; Bett et al., 2009). For example, in R6/2 mice, which express an N-terminal fragment of polyQ huntingtin, the proteasome was significantly impaired in synapses of the striatum and in cultured neurons (Wang et al., 2008), but a similar effect was not seen at a global level within the brain (Bett et al., 2009), suggesting compartment specific differences. In support of this idea, reporter systems have demonstrated that proteasome activity is markedly lower in neurons compared to glia, potentially making them more sensitive to functional changes. Similarly, proteasome activity is lower in neuronal processes compared to the soma, and lower in the nucleus than the cytoplasm, reinforcing compartment specific differences that may impact vulnerability to pathogenesis (Tydlacka et al., 2009; Zhao et al., 2016).

Perhaps the most compelling evidence for proteasome dysfunction playing a key role in pathophysiology comes from successful attempts to modulate degradation through this pathway, resulting in reduced toxicity in disease models. Several excellent reviews have highlighted therapeutic strategies to promote degradation of mutant proteins through the proteasome, including Hsp90 inhibition (Waza et al., 2006; Reis et al., 2016), Hsp70 modulation (Pratt et al., 2014, 2015), and disaggregase enhancement (Shorter, 2017). Importantly, some of these strategies alleviate polyQ toxicity in the absence of severe off-target effects, suggesting that they warrant further exploration as therapeutic approaches for the broad treatment of polyQ disorders. These studies have spurred diverse therapeutic efforts which have been recently thoroughly reviewed (Esteves et al., 2016). The extensive evidence implicating the proteasome as a therapeutic target, as well as its dysfunction and mislocalization in polyQ diseases, highlight its importance in the pathophysiology of disease.

## Conclusions

Recent advances in our understanding of polyQ disease pathophysiology have shed light on several divergent pathways of toxicity downstream of the mutant protein (Mhatre et al., 1993; Chamberlain et al., 1994; Kazemi-Esfarjani et al., 1995; Irvine et al., 2000; McCampbell et al., 2000; Lieberman et al., 2002; Szebenyi et al., 2003; Morfini et al., 2006; Ranganathan et al., 2009; Kemp et al., 2011; Giorgetti et al., 2016; Rocchi et al., 2016). As a result, a major focus of the field has been on either preventing synthesis of mutant proteins using antisense oligonucleotides (Lieberman et al., 2014; Sahashi et al., 2015; Giorgetti et al., 2016) or enhancing degradation by leveraging the endogenous cellular machinery (Sittler et al., 2001; Adachi et al., 2003, 2007; Tokui et al., 2009; Wang et al., 2013; Silva-Fernandes et al., 2014). The latter approach highlights the importance of ongoing research into the pathways that disaggregate, ubiquitinate, deubiquitinate, and degrade mutant proteins. Potential therapeutic targets have been identified within all of these key processes, and highlight the possibility of leveraging existing cell machineries for therapeutic benefit. We propose that a combination of approaches is likely necessary to limit polyQ protein toxicity while minimizing off target effects of therapeutics. The many advances highlighted in this review, as well as countless others in the fields of mutant gene silencing and trophic factor stimulation provide an optimistic outlook toward the future of disease treatments for polyglutamine disorders.

## Author contributions

SN and AL co-authored this manuscript. SN is a student in the M.D.-Ph.D. program at the University of Michigan Medical School and a thesis student in AL's laboratory.

### Conflict of interest statement

The authors declare that the research was conducted in the absence of any commercial or financial relationships that could be construed as a potential conflict of interest.
